# Global Insights Into Spondylodiscitis: A Bibliometric Analysis of Four Decades of Research

**DOI:** 10.7759/cureus.89001

**Published:** 2025-07-29

**Authors:** Julius Gerstmeyer, Anna Gorbacheva, Clifford Pierre, Luke Jouppi, Mark Kraemer, Nicholas Minissale, Cameron Hogsett, Colin Gold, Stephen D Lockey, Thomas A Schildhauer, Amir Abdul-Jabbar, Rod J Oskouian, Jens Chapman

**Affiliations:** 1 Orthopedics and Trauma Surgery, BG University Hospital Bergmannsheil, Ruhr University, Bochum, DEU; 2 Neurosurgery, Seattle Science Foundation, Seattle, USA; 3 Neurosurgery, Swedish Neuroscience Institute, Seattle, USA; 4 Complex Spine Surgery, Swedish Neuroscience Institute, Seattle, USA; 5 Orthopedic Surgery, University of Virginia, Charlottesville, USA; 6 Orthopedics and Spine Surgery, Swedish Neuroscience Institute, Seattle, USA

**Keywords:** bibliometric, spinal infection, spinal osteomyelitis, spine surgery, spondylodiscitis

## Abstract

The diagnosis and management of spondylodiscitis (SD) remain challenging for clinicians due to its insidious onset and nonspecific clinical presentation. Patients often have multiple comorbidities, requiring a multidisciplinary approach that may involve both nonsurgical and surgical interventions. This bibliometric study aims to analyze global research output on SD, exploring publication trends, citation patterns, contributing countries and journals, and commonly associated keywords.

Data were collected from the Clarivate Analytics Web of Science (WoS) database on July 22, 2024, using the search terms “Spondylodiscitis” OR “Spondylodiskitis” OR “vertebral osteomyelitis” OR “spine osteomyelitis” OR “spinal osteomyelitis” OR “osteodiscitis” OR “osteodiskitis” OR “discitis” OR “diskitis”. A total of 4,363 relevant publications were identified, spanning the years 1982 to 2023. Descriptive and quantitative analyses were conducted, including keyword network visualizations.

There was an almost 15-fold increase in publication volume, from 18 publications in 1982 to 269 in 2023. Citation frequency followed a similar trajectory, rising from a single citation in 1982 to 7,148 in 2023. The United States, Germany, and France accounted for over 50% of the total research output, with *Spine* identified as the most prolific journal. Commonly co-occurring keywords included “endocarditis” and “staphylococcus aureus,” as revealed by network visualization analysis.

In conclusion, SD is a complex condition requiring multidisciplinary care. This bibliometric review demonstrates significant growth in both research volume and citation impact, particularly from high-income countries. Notably, keyword mapping revealed an underrepresentation of comorbidity-related terms in recent literature, indicating a potential gap in current academic discourse.

## Introduction and background

Spondylodiscitis (SD) remains a significant clinical challenge due to its insidious onset and nonspecific presentation. This infection of the spinal column, characterized by varying degrees of vertebral body destruction, with or without intervertebral disc involvement, can be further categorized into de novo cases, hematogenous spread, or secondary forms resulting from extrinsic pathogen inoculation, such as iatrogenic causes [1,2]. The subtle presentation, often limited to vague symptoms like fever, back pain, and occasional neurological deficits, frequently delays diagnosis and the initiation of effective treatment [3]. Established risk factors include immunocompromised states, morbid obesity, systemic steroid use, hepatic dysfunction, renal dialysis, malnutrition, diabetes mellitus, intravenous (IV) drug use, and older age [2]. Significant global variation persists in the understanding and management of both pyogenic and non-pyogenic spinal infections, with reliable, durable epidemiologic comparisons remaining scarce [4-6]. The global incidence of SD appears to be increasing, as demonstrated by a reported rise to 14.4 cases per 100,000 inhabitants over a 10-year period in Germany [4]. While structured approaches such as the Spinal Infection Treatment Evaluation (SITE) score have been proposed for risk stratification, anecdotal and institution-specific decision-making still predominates [7,8]. Bibliometric analysis of publication volume, citation trends, and evolving research topics offers valuable insight into historical and emerging priorities in SD research. This study aims to provide a comprehensive overview of SD-related research output, with a focus on general publication trends, subspecialty developments, and their geographic distribution.

## Review

Methods 

The Clarivate Analytics Web of Science (WoS) database was utilized for data collection on July 22, 2024, using the following search strategy: TS = ("spondylodiscitis" OR "spondylodiskitis" OR "vertebral osteomyelitis" OR "spine osteomyelitis" OR "spinal osteomyelitis" OR "osteodiscitis" OR "osteodiskitis" OR "discitis" OR "diskitis") AND DT = ("Article" OR "Review") AND PY = (1900-2023). Only original articles and review articles were included; other publication types were excluded. Records from 2024 were omitted due to incomplete annual data. The full dataset and citation information were exported, and duplicate entries were identified and removed using DOI filtering in Microsoft Excel (Microsoft Corp., Redmond, WA, USA). Descriptive and quantitative analyses were performed using Excel (Version 16.86). For bibliometric network analysis, including keyword co-occurrence, VOSviewer software (Version 1.6.20, Centre for Science and Technology Studies, Leiden University, The Netherlands) was employed. Keywords appearing in five or more records were included using default visualization settings. All rankings presented include the top 10 entries per category.

Results

Publication Trends

**Figure 1:**
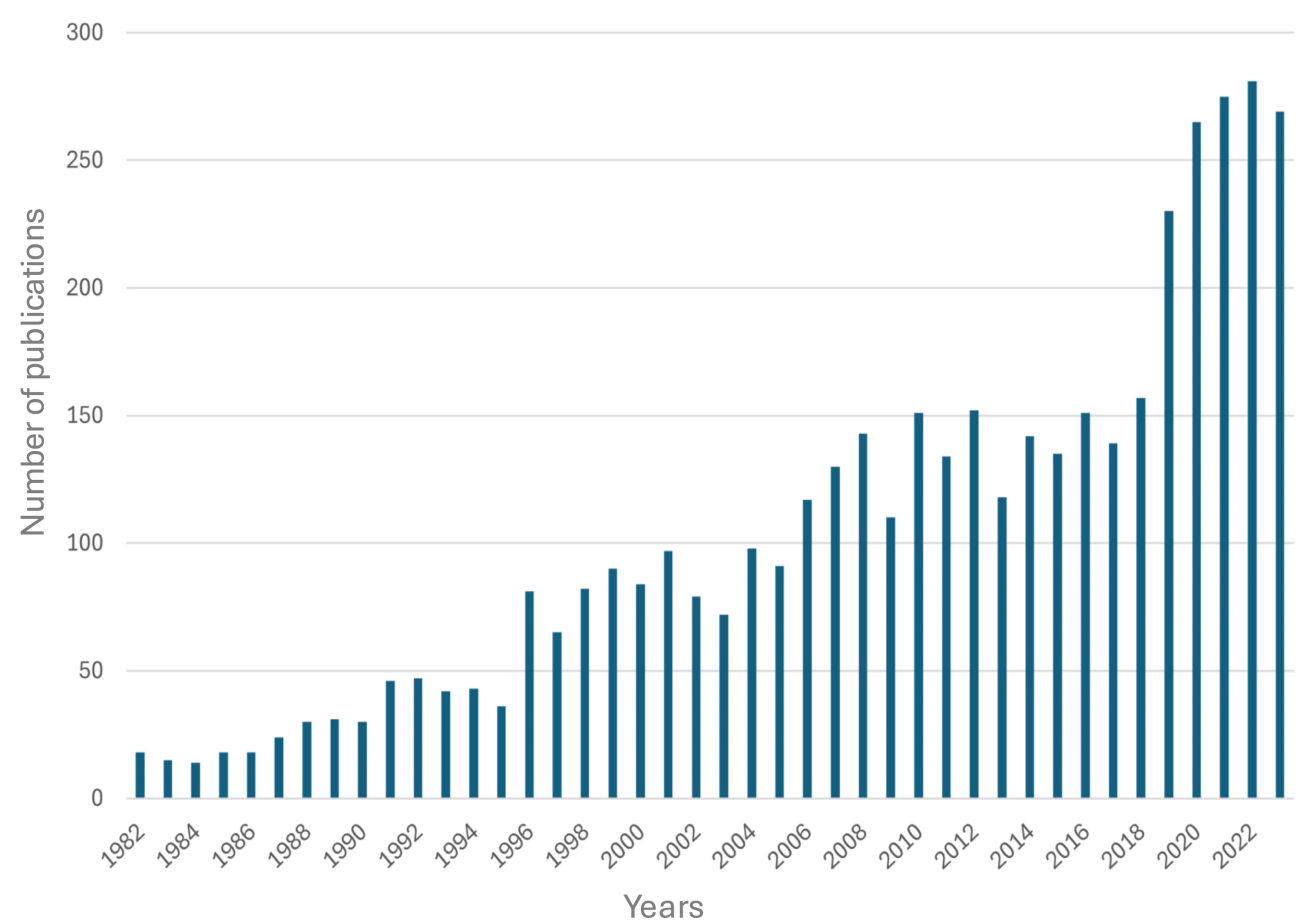
Number of publications per year related to spondylodiscitis.

A total of 4,363 publications met the inclusion criteria, of which 12.3% (n = 570) were review articles. The publication trend analysis (Figure 1) spanned from 1982 to 2023. During this period, the number of annual publications increased nearly 15-fold, from 18 in 1982 to 269 in 2023, representing an approximate 1,500% rise. A consistent year-over-year growth in publication output was observed. The largest single-year increase occurred in 2019, with the number of publications rising from 157 in 2018 to 230, marking a surge of 87 articles.

Citations

**Figure 2:**
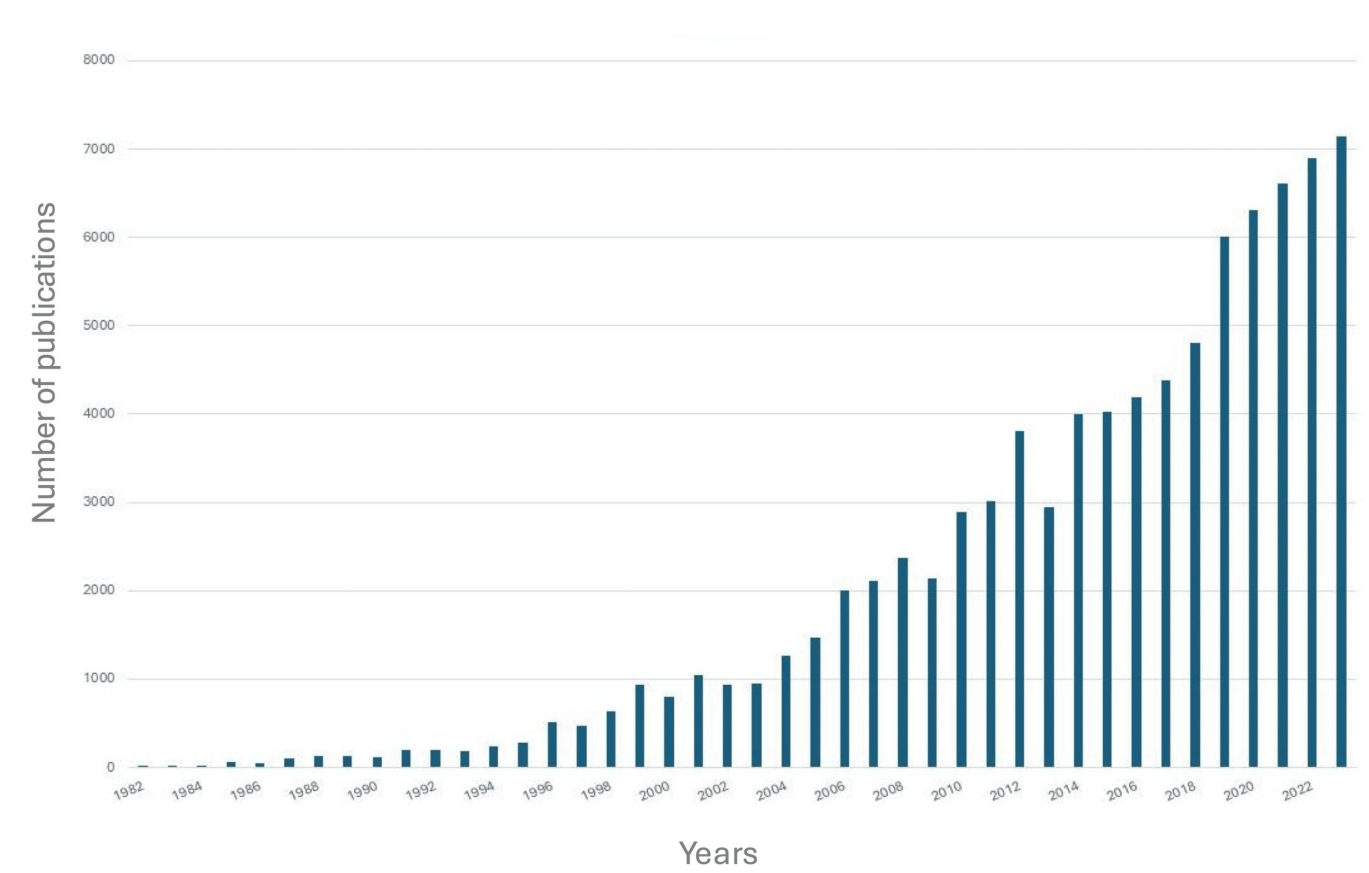
Number of citations per year from 1982 to 2023.

The citation analysis (Figure 2) demonstrated a marked increase in total citations over time, rising from a single citation in 1982 to 7,148 citations by 2023. The most significant year-over-year growth occurred between 2018 and 2019, with citations increasing from 4,800 to 6,011, paralleling the notable rise in publication output during the same period.

Countries

**Figure 3:**
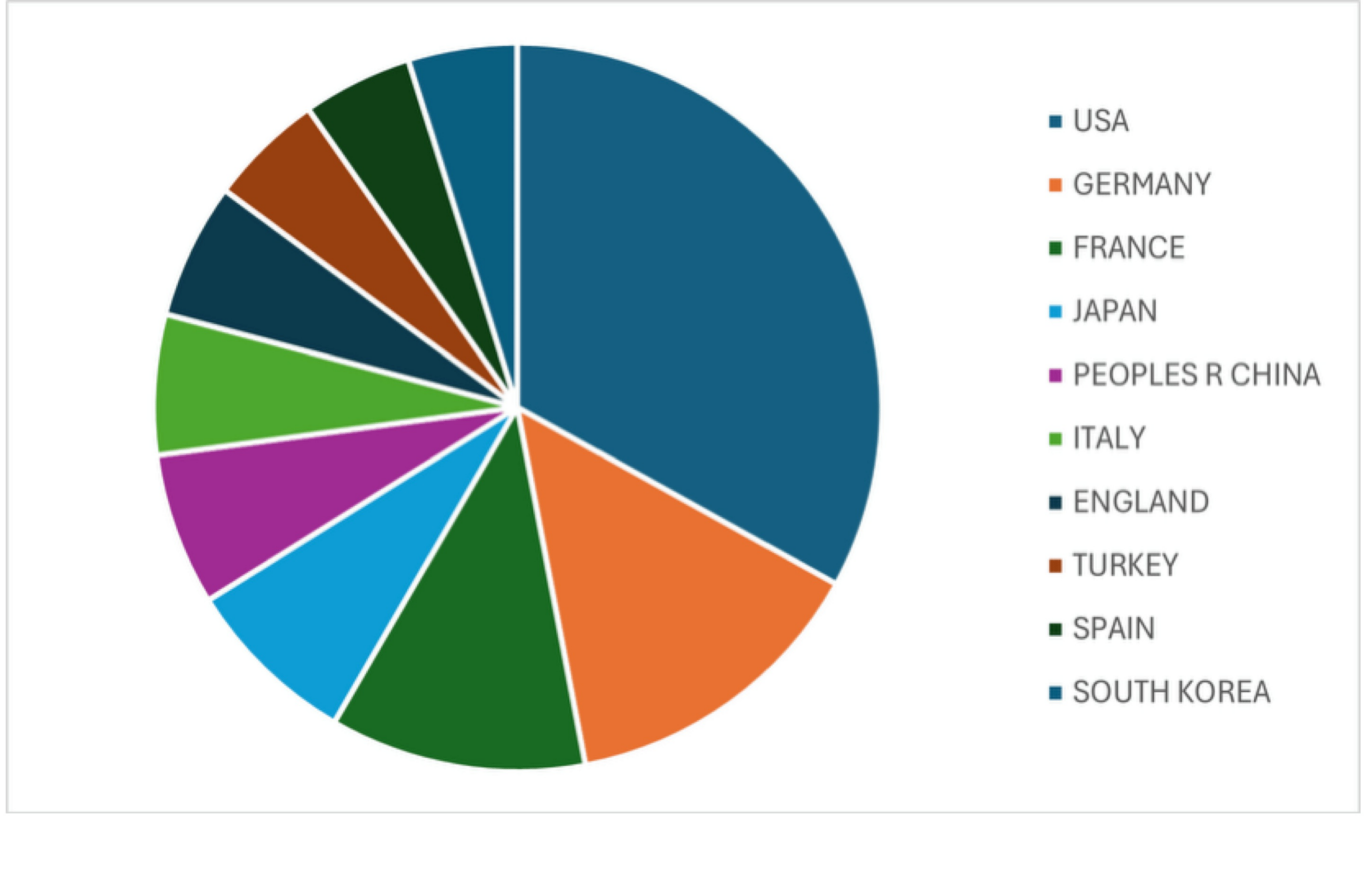
Top 10 publishing countries.

In the analysis of the top 10 publishing countries (Figure 3), the United States ranked first with 1,100 publications, accounting for approximately 25% of the global academic output related to SD. Germany followed with 464 publications (11%), and France ranked third with 379 (9%). Other notable contributors included Japan (6%), the People’s Republic of China (5%), Italy (5%), the United Kingdom (5%), Turkey (4%), Spain (4%), and South Korea (4%). Notably, no African countries appeared among the top 10 publishing nations.

Journals

**Figure 4:**
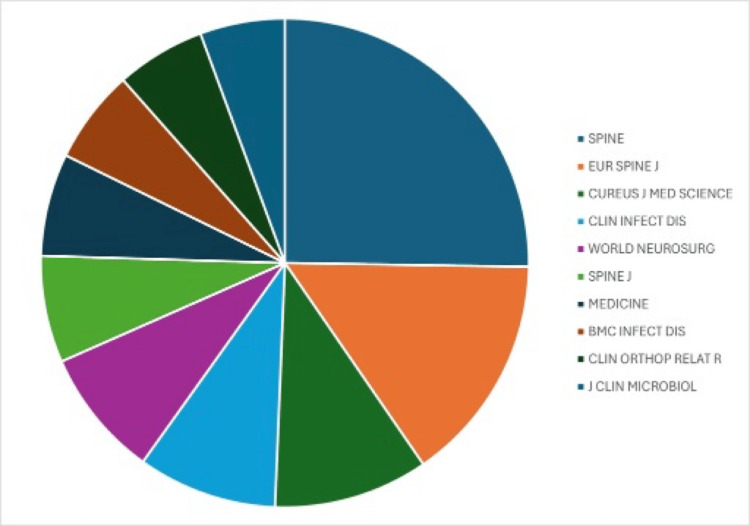
Top 10 publishing journals.

A total of 968 journals have published articles on the topic of SD, as illustrated in Figure 4. The most prolific journal was *Spine*, with 175 publications. This was followed by the *European Spine Journal*, which accounted for 105 publications. *Cureus Journal of Medical Science* ranked third with 71 articles. Other notable contributors included *Medicine*, which ranked seventh, and the *Journal of Clinical Microbiology*, which was tenth with 39 publications.

Keyword Network Visualizations

**Figure 5:**
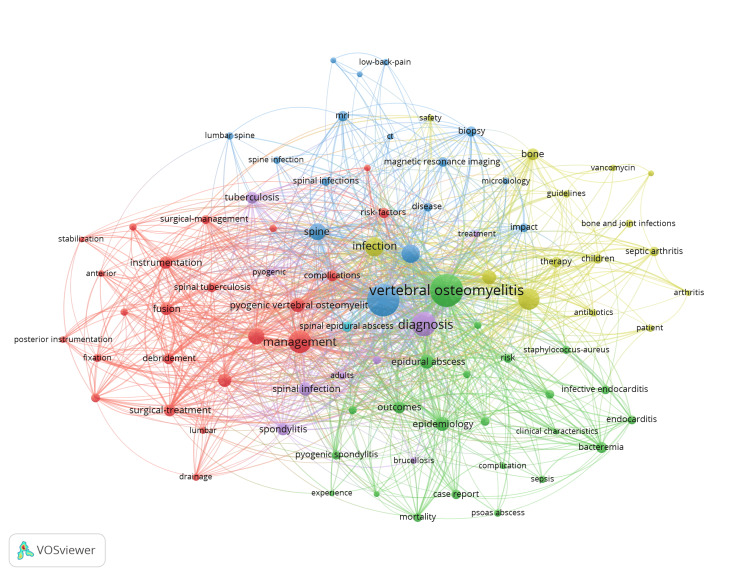
Keyword network visualization of all-time data. The size of the nodes related to each keyword reflects its frequency of occurrence in the literature. Links between words represent the frequency of their co-occurrences in individual publications. Thematic clusters are represented in the colors of the groups. Image Credit: Authors' original creation.

**Figure 6:**
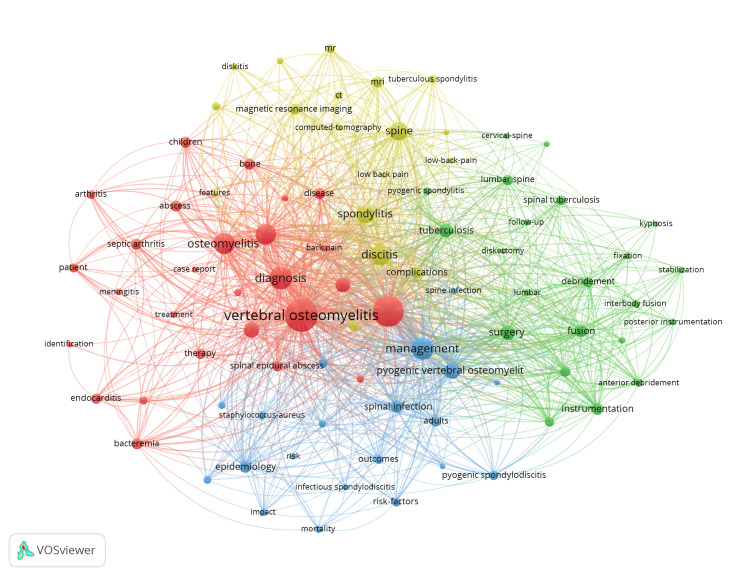
Keyword network visualization based on the last five years of data. The size of the nodes related to each keyword reflects its frequency of occurrence in the literature. Links between words represent the frequency of their co-occurrences in individual publications. Thematic clusters are represented in the colors of the groups. Image Credit: Authors' original creation.

The keyword co-occurrence network visualization for all time (Figure 5) shows that terms related to "management" and "diagnosis" appear in close proximity to "SD," indicating their central role in the research focus. Among comorbidities, "endocarditis" remained a consistently represented term, while others were notably underrepresented. From a microbiological standpoint, "tuberculosis" and "staphylococcus aureus" were the most prominent pathogens associated with SD. Interestingly, "meningitis" emerged as a relevant keyword in the five-year keyword analysis (Figure 6), suggesting a recent research trend. Additionally, while surgical treatment featured prominently in the all-time analysis, its relative prominence decreased in the recent five-year data.

Discussion

The rising incidence of SD continues to challenge healthcare professionals globally. Despite advances in diagnostic capabilities, the management of SD remains inconsistent and unstandardized across healthcare systems, with reported mortality rates as high as 14% [8-10]. The disease’s nonspecific and often subtle clinical presentation can delay diagnosis and the timely initiation of appropriate treatment [3]. Analyzing trends in academic publications related to SD may offer valuable insights and help address the ongoing challenges in its management.

Our publication trend and citation analyses demonstrated growing interest in SD, with a 15-fold increase in overall publications, from 18 in 1982 to 269 in 2023, and a rise in citations from 1 to 7,148 over the same period. This surge likely reflects increased recognition of the disease, possibly driven by an aging and increasingly comorbid population [11]. The body of research also highlights the ongoing lack of consensus on treatment strategies, which remain largely anecdotal and vary across healthcare systems, rather than being guided by standardized, evidence-based algorithms. Among the top 10 publishing countries, the United States, Germany, and France accounted for around 45% of all SD-related publications. This may be due to the concentration of well-funded academic institutions and greater government support for research in these countries [12]. Overall, North America and Europe contributed 27% and 46% of the total publications, respectively, amounting to 73%, while the remaining 27% came from the rest of the world. Notably, no countries from Africa or South Asia appeared in the top 10, despite the high prevalence of pathogens such as *Mycobacterium tuberculosis* and *Brucella *spp. in those regions [13-15]. This geographic gap in the literature raises concerns about selection bias, as it may not capture the full range of diagnostic and treatment challenges faced in areas with different pathogen profiles. The 4,363 publications included in our analysis were spread across 968 journals. The top three were *Spine*, *European Spine Journal*, and *Cureus Journal of Medical Science*, with 175, 105, and 71 publications, respectively. While spine-focused journals dominated, the inclusion of general medical journals such as *Medicine *within the top 10 underscores the condition’s multidisciplinary relevance. Overall, the findings highlight persistent concern around comorbidities in SD and reflect the broad academic interest in improving its management.

In our keyword co-occurrence network visualizations, the terms “pyogenic” and "staphylococcus aureus" appeared frequently and in close proximity, as expected, since *S. aureus* is the most common pathogen implicated in SD, responsible for roughly half of non-tuberculous cases [16]. Tuberculosis was also featured prominently, consistent with its significant global burden and its association with 17%-40% of SD cases [17,18]. However, it was surprising that terms related to immunocompromised states, such as HIV or malnutrition, were not observed, despite their recognized role as risk factors for SD development [19]. In fact, comorbidities in general were notably underrepresented in the network visualization, apart from “endocarditis,” which appeared as a prominent keyword. This is noteworthy given the high relevance of endocarditis in hematogenous SD, especially in patients with IV drug use or those undergoing dialysis. Studies suggest that up to 30% of SD patients may also have endocarditis, highlighting the importance of its early recognition during workup [20]. Interestingly, “meningitis” emerged as a relevant keyword in the last five years, which may reflect growing research interest and better diagnostic detection. Although the co-occurrence of SD and meningitis remains relatively rare, reported in 6.4% of cases in a cohort of 469 patients, it may be increasingly identified with improved diagnostic tools and heightened clinical awareness [21]. As diagnostic methods advance, co-existing infections such as endocarditis, meningitis, and epidural abscesses may be detected more frequently, particularly in cases involving hematogenous spread.

Bibliometric analyses are valuable tools for uncovering knowledge gaps and guiding future research directions. By using automated keyword searches across thousands of publications, these methods allow researchers to analyze large datasets efficiently without manually reviewing each entry. However, these tools have limitations. Results must be interpreted cautiously, as inclusion of both original and review articles may inflate publication and citation counts [22]. Still, bibliometric studies can provide a useful snapshot of current clinical understanding and highlight areas that warrant further investigation.

## Conclusions

SD remains a challenging spinal pathology, despite advancements in diagnostic techniques and management over time. Our analysis demonstrated a steady increase in both publications and citations, with most contributions originating from high-income countries. Research on SD spans multiple surgical and medical specialties, reflecting its complex nature as both a spinal and infectious disease that requires multidisciplinary management. Notably, the keyword network visualization revealed an underrepresentation of comorbidities in recent academic literature, highlighting a potential area for future research.
